# Validation of the German Relationship‐Obsessive Compulsive Inventory: Testing the Factorial Structure, Measurement Invariance, and External Validity

**DOI:** 10.1002/jclp.70024

**Published:** 2025-08-08

**Authors:** Kay Brauer, Lara Borchardt

**Affiliations:** ^1^ Department of Psychology Martin Luther University Halle‐Wittenberg Halle (Saale) Germany

**Keywords:** attachment styles, personality pathology, relationship obsessive compulsive disorder, Relationship Obsessive Compulsive Inventory, romantic relationships

## Abstract

**Objectives:**

Relationship obsessive compulsive disorder (ROCD) describes intrusive thoughts and compulsive behaviors (e.g., reassurance seeking, monitoring feelings) regarding one's romantic relationship. The Relationship Obsessive Compulsive Inventory (ROCI) is the standard instrument to assess ROCD expressions. In our study, we tested the reliability and validity of the German ROCI.

**Methods:**

We analyzed data from two independent nonclinical samples comprising 409 and 248 partnered individuals to expand the knowledge of the psychometric properties, factorial structure, and external validity of the German‐language ROCI.

**Results:**

Factor analyses supported the 3‐factor model, but we did not find evidence for models assuming a total score (unidimensional, bifactor‐, and second‐order models). Further, we found scalar measurement invariance between men and women. The reliabilities are satisfying (0.77–0.88). Finally, we localized the ROCI into systems of romantic attachment, personality pathology, and relationship satisfaction and found that ROCD is characterized by insecure attachment, low satisfaction, and inclinations to negative affectivity. The ROCI showed incremental validity when predicting relationship satisfaction beyond attachment.

**Conclusions:**

The German ROCI is psychometrically sound, and we recommend its application to assess expressions of ROCD in German‐speaking samples, and, thus, to study the prevalence and consequences of ROCD in German‐speaking countries and regarding cross‐cultural comparisons. We discuss limitations (e.g., lack of longitudinal data) and future directions (e.g., dyadic studies).

## 1 Introduction

Doron and colleagues (2012a, 2012b, 2014) introduced the concept of relationship obsessive compulsive disorder (ROCD), which describes frequently doubting and thinking about the “rightness” of the relationship and the partner. In addition to the cognitive component, those high in ROCD experience the need to repeatedly checking one's feelings towards the partner, comparing the partner and relationships to standards that might be derived by observing actual other couples or stereotypes that might be activated by, for example, observing a fictitious couple and relationship (e.g., in a movie; Doron et al. 2014), and seeking reassurance that their partner and current relationship are a good fit for them. In addition to questioning relationship‐centered aspects, ROCD can also be focused on the partner (e.g., perceptions and obsessions about their flaws; Doron et al. 2012a). ROCD is positively related to generalized OCD, but there is robust evidence that they are related but yet distinct phenomena (e.g., Doron et al. 2012b, 2016; Fernandez et al. 2021). Tinella and colleagues (2024) reported that 22% of their sample of 659 emerging adults show elevated ROCD symptoms. Since the introduction of ROCD to the field, numerous studies have contributed to understand its consequences for individuals and couples, its distinction from OCD as well as testing approaches to reduce ROCD (e.g., Cerea et al. 2020; Doron and Derby 2017; Gorelik et al. 2023). Correlates of ROCD include experiencing heightened levels of stress, lower relationship satisfaction, and jealousy to name but a few (e.g., Doron et al. 2012b, 2014; Kılıç and Altınok 2021).

The standard instrument to assess expressions of relationship‐centered symptoms of ROCD is the *Relationship Obsessive Compulsive Inventory* (ROCI; Doron et al. 2012b). To our knowledge, a German‐language translation of the ROCI is not available and no study has yet examined ROCD in German‐speaking countries. Hence, there is currently no available instrument to assess ROCD in German‐speaking countries. Testing the German translation of the ROCI will allow researchers and practitioners to extend the study of ROCD to German‐speaking countries and, building the foundation for research on ROCD (e.g., dyadic studies) in German‐speaking samples as well as cross‐cultural research (e.g., comparisons of findings across countries and multi‐centric studies) with regard to, for example, replicating and testing the invariance of prior findings in German‐speaking countries, testing potential treatment options, and allowing to assess ROCD expressions in clinical practice. Considering the available evidence on consequences of ROCD, such as maladaptive beliefs about relationships that affect the well‐being of those with high ROCD expressions (e.g., Doron et al. 2016), we argue that ROCD is an important phenomenon for understanding how people experience their relationship. We aimed to narrow this gap in the literature by testing a German translation of the ROCI and examining its reliability and factorial validity in two independently collected samples of partnered individuals, including the analysis of measurement invariance across gender. Further, we examined its external validity by replicating associations with attachment styles and relationship satisfaction, and by localizing the ROCI scores in the dimensional system of personality pathology (Krueger et al. 2012). We expect that providing the means to assess ROCD in German‐speaking countries will extend the knowledge about ROCD and its consequences across countries and languages.

### 1.1 The Relationship Obsessive Compulsive Inventory

Doron et al. (2012b) introduced the Relationship Obsessive Compulsive Inventory (ROCI), a 12‐item self‐report questionnaire that assesses expressions of ROCD. Factor analyses from two samples (*N* = 329 and 179) showed that the items are assigned to three correlated factors, *love for partner*, *relationship “rightness*,*”* and *being loved by the partner* (model fit: RMSEAs ≤ 0.094, CFIs = 0.96, SRMRs = 0.04). The internal consistencies are good (≥ 0.80). Doron et al. suggested that a total score can be employed in addition to the scale scores. While a unidimensional factor model did not fit the data (RMSEA = 0.14, SRMR = 0.06, CFI = 0.90), the authors argued that a total score can nevertheless be assumed because the fit of a second‐order model is equal to a correlated‐factor model. This notion has been adopted in later studies using only the ROCI total score. To our knowledge, no study has yet re‐examined the notion of a general factor that translates to a total score, especially by testing a bifactor model that assumes that items are loaded by a general factor *and* their specific scale factor.

The external validity of the ROCI has been demonstrated by Doron et al. (2012b) by testing associations with OCD, relationship satisfaction and romantic attachment, as well as measures of self‐esteem, depression, anxiety, and stress. Overall, their findings showed the expected correlations without indicating redundancy. Also, the ROCI showed incremental validity when predicting relationship satisfaction over and beyond attachment. Since the introduction of the ROCI, findings on the nomological and predictive validity from clinical and nonclinical samples have provided additional evidence for its reliability and validity (e.g., Doron et al. 2016; Gorelik et al. 2023; Szepsenwol et al. 2016; Tinella et al. 2023). Also, the ROCI has been translated to languages such as Turkish and Italian (Melli et al. 2018; Trak and İnözü 2017), showing that the three‐factorial structure replicated well, indicating robust internal consistencies, and support the criterion validity, thus, allowing to extend the research on ROCD from Hebrew‐ and English‐speaking samples to other countries.

### 1.2 The Present Study

We aimed to examine the German‐language version of the ROCI by investigating its psychometric properties and structural and external validity. Moreover, this is the first study to systematically investigate competing measurement models that are assumed in the literature and also examine the invariance of the best fitting across men and women. To ensure the stability of the findings on the psychometric properties, we collected and analyzed data from two independent samples consisting partnered individuals. In the following, we describe how we examined the reliability and validity of the German translation of the ROCI.

#### 1.2.1 Structural Validity

We tested the structural validity of the German ROCI using Confirmatory Factor Analyses (CFA) and examined four models that potentially represent the ROCI's structure according to the literature. First, we examined the standard model assuming three correlated factors, originally introduced by Doron et al. (2012b). Secondly, we examined three alternative models that reflect the assumption of a total score by means of (a) an unidimensional model assuming that a single factor accounts for the interrelations between the ROCI items, (b) a second‐order factor model with a general factor being made up of the three correlated specific factors, and (c) a bifactor model that assumes that each item is loaded by a specific factor *and* a general factor. We aimed to identify the model that provides the best fit to the data in terms of model fit but also stable and interpretable loading patterns across samples. Also, prior research used regular maximum‐likelihood estimators for factor analyses that do not account for the 5‐point rating format the ROCI utilizes. Accordingly, we analyzed the data using the WLSMV estimator, which has been introduced to deal with data generated from Likert‐type rating scales (Brauer et al. 2023). We expect that our findings will contribute to the knowledge about the measurement model of the ROCI.

After we identified the best‐fitting model, we tested the measurement invariance of the ROCI measurement model between men and women. Although previous studies have found negligible gender differences in the ROCI scores (e.g., Doron et al. 2012a, 2012b; Kılıç and Altınok 2021; Tinella et al. 2023, 2024), it remains untested, to our knowledge, whether the ROCI's measurement models are invariant across gender. Expanding the knowledge about measurement invariance is important to understand whether manifest scores are comparable between men and women and imply whether differences might be traced back to differences in the measurement and response behaviors. If we find support for measurement invariance, it can be assumed that gender differences can be interpreted in a meaningful manner (Chen 2007).

#### 1.2.2 Psychometric Properties and Reliability

After identifying the best‐fitting measurement model, we conducted item‐ and scale analyses (i.e., item difficulties and item‐total correlations) to examine the psychometric properties of the German ROCI. We tested the reliability in terms of internal consistency.

#### 1.2.3 Nomological Validity

We tested the external validity of the German ROCI on basis of the data from Sample 2. We localized the German ROCI in systems of attachment styles, maladaptive personality traits in the sense of the recommended dimensional system of personality disorders according to the DSM‐5 (Krueger et al. 2012), and relationship satisfaction. While we expected to replicate findings on associations with satisfaction and attachment from prior research (e.g., Doron et al. 2012a, 2012b; Kılıç and Altınok 2021), this is the first study that localized the ROCI facets in broad domains of personality pathology. Overall, we expected to find similar relationships between the study variables as reported in prior research.

Attachment styles describe internal working models of how people perceive, feel in, and experience close relationships (Hazan and Shaver 2017). Individual differences in attachment are described by two orthogonal dimensions, namely, anxiety (i.e., feelings of insecurity in one's relationship and fear of abandonment while longing for security) and avoidance (i.e., dismissing relationships and one's partner, experiencing discomfort from closeness). Attachment styles are predictive of numerous variables in relationships (e.g., current and retrospective singlehood, relationship dissatisfaction, and low positive psychological functioning; e.g., Brauer and Proyer 2020; Brauer et al. 2020; Feeney 1999; Fraley 2019; Neumann et al. 2007). The ROCI scales show positive correlations with both attachment styles, with correlations between 0.24 and 0.35 (Doron et al. 2012a, 2012b). However, the findings should be interpreted cautiously considering that attachment was assessed with a very brief form (12 instead of 36 items) of the Experiences in Close Relationships instrument, yielding low reliabilities of 0.58 (avoidance) and 0.72 (anxiety) in their study. Hence, assessing the relationships between the ROCI and attachment using the full 36‐item attachment measure will reduce the potential influence of measurement error and provide reliable correlation estimates.

Relationship satisfaction is the subjective experience and evaluation of one's romantic relationship (Hahlweg, 1996). The ROCI scores correlate negatively with relationship satisfaction, with coefficients between −0.39 and −0.61 (Doron et al. 2012a, 2012b; Kılıç and Altınok 2021). Further, the ROCI scores yielded incremental validity by predicting relationship satisfaction above and beyond romantic attachment styles, depression, anxiety, and self‐esteem in stepwise regression analyses, providing an increment of Δ*R* ^2^ = 0.03. We expected to find similar results when predicting relationship satisfaction after controlling for attachment.

Finally, we localized the ROCI scales in the system of maladaptive personality traits of the DSM‐5 (Krueger et al. 2012), that describes five broad dimensions representing major domains of personality pathology. These include Detachment (inclinations to anhedonia and depressiveness), Negative Affectivity (i.e., anxiousness and emotional lability), Antagonism (i.e., showing callousness and hostility), Psychoticism (i.e., eccentricity and holding unusual beliefs and experiences), and Disinhibition (e.g., distractibility and impulsivity). This dimensional approach to personality pathology has expanded the understanding of personality disorders and reduced diagnostic problems of categorical approaches to personality pathology by considering individuals’ fine‐grained expressions in the five domains (Fowler et al. 2017). Recently, Esfahan et al. (2024) used item‐level network analyses to examine the overlap between the five domains of maladaptive traits and ROCD. They found that only the cluster of items representing negative affect accounted for variance of the ROCI items. To our knowledge, this is the first study examining the correlations between the domains of personality pathology and ROCD on the scale level. Understanding the localization of ROCD in the broader domains of maladaptive traits contributes to learning more about how ROCD relates to clinically relevant traits describing patterns of dysfunctional emotions, thinking, and behaviors that are relatively stable across time and situations.

Taken together, our findings will extend the knowledge regarding the ROCI's factorial structure, measurement invariance, and external validity. We will address the latter by localizing the ROCI in systems of attachment, relationship satisfaction, and personality pathology as well as testing the incremental validity of the ROCI.

## 2 Method

### 2.1 Participants and Procedure

Sample 1 contained 409 partnered participants aged between 17^1^ and 64 years (*M* = 26.1, SD = 7.1). Their average relationship duration was 3.9 years (ranging between 1 month and 52 years). Most (79.2%) identified as women, 19.8% as men, and 1% indicated “other.” Their educational status was high because more than half (62.8%) held the high school diploma qualifying to attend university (“Abitur”), 28.1% held a university degree, 7.1% completed vocational training, and the remainder held a high school diploma.

Sample 2 comprised 248 partnered participants aged between 17 and 73 years (*M* = 25.1, SD = 8.1). On average, the relationship duration was 4.0 years (ranging between 1 month and 36 years). The majority identified as women (80.1%), 19.0% were men, and 0.8% indicated “other” as their gender. Educational status was high, with 67.7% holding the Abitur, 25.7% held a university degree, 5.6% completed vocational training, and remaining participants finished high school.

We collected the data online between November 2022 and May 2023 (Sample 1) and January and March 2024 (Sample 2). We hosted the link to the online questionnaire on the authors’ department's website and advertised the study as research on personality traits in romantic relationships on‐campus in message boards. Inclusion criteria were speaking German and being currently in a romantic relationship. There was no financial compensation for participation, but psychology students could earn course credit.

We downloaded only complete data sets from SoSciSurvey, where participants were required to answer all items or were reminded of any missing responses by the software. Accordingly, the data sets did not contain missing values. We examined the validity of the responses with SoSciSurvey's time‐based index of valid responses (see Leiner 2019) and additionally checked the data sets for irregular response behaviors (e.g., choosing identical responses to item sets). We did not find irregularities that would point to invalid responses.

### 2.2 Instruments

Participants of both samples completed the German translation of the ROCI. Our translation of the ROCI to German was based on the translation—back‐translation procedure recommended by the International Test Committee (International Test Commission. 2017) guidelines. Two bilingual psychologists who are experts as defined by the ITC (i.e., having knowledge about the languages involved, the cultures, experience in test development, and the research domain of romantic relationships) translated the items independently of each other and discussed refinements before back‐translation. In an additional round, we also compared our translation with computer‐aided translations using Google Translate and the DeepL software, respectively. Doing so showed that they converged well. Further, we provided the questionnaire for a pre‐test to five lay persons and asked them to provide feedback, which showed that no misunderstandings for the item content existed. As in the original version, the response format is a 5‐point rating scale with the anchors 1 = *not at all* and 5 = *very much*. Participants of Sample 2 completed the measures described below in addition to the German‐language ROCI. The German translation of the ROCI is provided in Supplemental Table A.

We assessed romantic attachment styles with the Experience in Close Relationships questionnaire (ECR; Brennan et al. 1998; German: Neumann et al. 2007) that allows to assess the orthogonal dimensions of attachment anxiety and attachment avoidance. The ECR comprises 36 items and participants give their responses on a 7‐point rating scale (1 = *does not apply*; 7 = *fully applies*). Sample items are “I'm afraid that I will lose my partner's love” (anxiety) and “I prefer not to show a partner how I feel deep down” (avoidance). There is robust evidence for the good psychometric properties and validity of the German‐language version of the ECR (Brauer and Proyer 2025; Neumann et al. 2007). In line with earlier findings on the German ECR's internal consistencies, with αs ≥ 0.83, anxiety and avoidance yielded coefficients of 0.88 and 0.89 in the present study.

We used the Personality Inventory for the DSM‐5‐Brief Form (PID‐5‐BF) by Zimmermann et al. (2012; German: Zimmermann et al. 2014) to assess expressions in maladaptive personality traits. The PID‐5‐BF assesses five broad domains of personality pathology with five items each, namely, negative affectivity (e.g., “I get emotional easily, often for very little reason”), antagonism (e.g., “It's no big deal if I hurt other peoples’ feelings”), detachment (e.g., I often feel like nothing I do really matters”), disinhibition (e.g., “People would describe me as reckless”), and psychoticism (e.g., “Things around me often feel unreal, or more real than usual”). The brief form of the PID‐5 converges well with the full 220‐item instrument and studies of its psychometric properties and validity concluded that the PID‐5‐BF is well suited to provide a brief assessment of the major domains of maladaptive personality traits (Anderson et al. 2018; Gomez et al. 2020). In line with earlier research, we found internal consistencies of 0.63 (Negative Affectivity), 0.66 (Detachment), 0.70 (Antagonism), 0.67 (Disinhibition), and 0.72 (Psychoticism). While the PID‐5 and its brief form were initially administered to participants ≥ 18 years of age, there is robust evidence that it is suitable for 17‐year‐olds (Koster et al. 2020).

We assessed relationship satisfaction with the 9‐item Short Relationship Questionnaire (SRQ; Kliem et al. 2012). An example item is “My partner tells me that they love me.” Participants give their responses on a 4‐point Likert scale (1 = *never/rarely*, 4 = *always*). An additional single item assesses overall satisfaction with one's relationship on a 6‐point rating scale (1 = *very unhappy*; 6 = *very happy*). The instrument is frequently used in relationship research of German‐speaking samples and there is robust evidence for its reliability (α = 0.84) and structural and external validity (e.g., Kliem et al. 2012; Körner and Schütz 2025). In the present study, the internal consistency was 0.82.

### 2.3 Data Analysis

#### 2.3.1 Factor Analysis

We computed CFAs in Mplus 8.8 (Muthén and Muthén 1997–2019) using the WLSMV estimator to account for the ordinal nature of the data (see Brauer et al. 2023). We tested four factor models that have been discussed in the literature: (1) a three‐dimensional model reflecting the three correlated scales of the ROCI as suggested by Doron et al. (2012a, 2012b) and (2) a unidimensional model reflecting the notion that the measurement model of the ROCI would be best modeled as a single factor that translates into an exclusive total score, (3) a second‐order model with three correlated factors that are loaded by a general factor, and (4) a bifactor model in which the items are loaded by three specific factors (reflecting the scales) *and* the general factor (Reise. 2012). Since this is the first study testing a bifactor model of the ROCI, we used the exploratory bifactor model because it allows to examine the loading structure regarding potential cross‐loadings. Considering that traditional cut‐offs for model fit criteria (RMSEA ≤ 0.08, and CFI and TLI ≥ 0.95, SRMR ≤ 0.08 indicating model fit; Asparouhov and Muthén 2018; Hu and Bentler 1998) are not validated for the WLSMV estimator and, thus, work only as rough rules of thumb (Moshagen and Musch 2014) and since we used a comparative approach, we aimed to identify the model with the best fit *and* a replicable and reasonable loading pattern as the best candidate to represent the measurement model of the German ROCI.

#### 2.3.2 Measurement Invariance

We examined the invariance of the ROCI measurement models between men and women with multi‐group confirmatory factor analysis. We tested three models that differed in model constraints: First, the configural model assumes that the number of factors is the same in the subsamples of men and women; second, the metric model constrains the factor loadings to be equal between men and women; thirdly, scalar invariance additionally assumes invariance of item intercepts. When model fit does not robustly decrease after inducing the consecutive constraints, measurement invariance is assumed. We used Chen. (2007) recommendations when evaluating the change in model fit between models. We rejected metric invariance when ΔCFI ≥ 0.010 *and* ΔRMSEA ≥ 0.015 (or ΔSRMR ≥ 0.030) and rejected scalar invariance when ΔCFI ≥ 0.010 *and* ΔRMSEA ≥ 0.015 (or ΔSRMR ≥ 0.010). We aggregated the data from Samples 1 and 2 for this analysis. To exclude the possibility of biases due to imbalance in the subgroup sample sizes, we followed recommendations by Yoon and Lai (2018) and have drawn a random subsample of women that matched the number of men to yield a 50:50 ratio of men and women. Hence, we analyzed a subsample of *n* = 256 participants.

#### 2.3.3 Reliability

We estimated the reliability by means of two measures of internal consistency, Cronbach's α and McDonald's ω. The latter has less strict assumptions and computes internal consistency based on factor loadings (Dunn et al. 2014). Further, we examined the corrected‐item total correlations for the items and considered them good when exceeding 0.40 (Zijlmans et al. 2018).

#### 2.3.4 External Validity

We computed bivariate Pearson correlations to examine the relations between the ROCI and our external measures. We interpret correlations ≥ 0.10, 0.20, and 0.30 as small, moderate, and large effect sizes (Funder and Ozer 2019). We examined the incremental validity of the ROCI in line with Doron et al. (2012a, 2012b) by testing the additional variance explained (change in *R* ^2^) when predicting relationship satisfaction after controlling for romantic attachment. Further, we examined the regression effect size *f* ^2^ that indicates a small effect when 0.02, near 0.15 as medium, and greater 0.35 as large effects (Cohen 1988). Therefore, we computed a blockwise regression, with attachment anxiety in Block 1 and the ROCI in Block 2.

Since the five maladaptive traits assessed by the PID‐5 are inter‐related (Anderson et al. 2018), we extended the correlation analyses between the ROCI and PID‐5 by computing stepwise regression analyses. This allowed us to examine the unique variance explained by the PID‐5 traits and to identify which of the PID‐5 traits robustly relates to the ROCI as well as computing the regression effect size *f* ^2^ for each step and PID‐5 trait as outlined above.

#### 2.3.5 Power

The sample sizes were sufficient to estimate robust correlation matrices and factor analyses (Moshagen and Musch 2014). We computed a power analysis (type = sensitivity) for the validity analysis in Sample 2 with G*Power (Faul et al. 2009), which showed that our sample size allowed to detect correlations ≥ 0.20 with 90% power and 5% type‐I‐error rate using two‐tailed tests of statistical significance. (https://osf.io/gf9yr/?view_only=10cd1ae37ad04889b4041b9f854620fc).

## 3 Results

### 3.1 Confirmatory Factor Analyses

We first examined unidimensional and three‐factorial measurement models. Our analyses showed that the assumption of a single factor did not fit the data well in both samples (RMSEAs ≥ 0.158, CFIs and TLIs ≤ 0.906, and SRMRs ≥ 0.10; see Table 1). The measurement model with three correlated factors fit comparatively well with the data and aligned with Doron et al. (2012b), yielding RMSEAs ≤ 0.107, CFIs ≥ 0.955, TLIs ≥0.942, and SRMRs ≤ 0.06 in both samples (Table 1). The comparison of the models showed that the three‐factor model yielded better model fit than the unidimensional model according to chi‐square difference tests of model comparison (Δχ^2^[3] > 267, *p*s < 0.001) and descriptive differences of the model fit indices (ΔRMSEA > 0.06, ΔCFI > 0.07, ΔTLI > 0.09). Adopting the three‐factor model showed that all loadings were robust (0.50 ≤ λ ≤ 0.73; all *p*s < 0.001; see Table 2 for all loadings). The factor correlations were 0.61/.72 (FI—FII), 0.32/.39 (FI—FIII), 0.59/.50 (FII—FIII) in Samples 1 and 2, respectively.

**Table 1:** Model Fit Indexes from Confirmatory Factor Analyses of Unidimensional and Three‐Dimensional Models, and Model Comparison (Δ).

	RMSEA	90%‐CI	CFI	TLI	SRMR	*χ* ^2^
Sample 1 (*N* = 409)						
Unidimensional	0.170	[0.159, 0.182]	0.880	0.853	0.11	692.94
Three‐dimensional	0.107	[0.095, 0.119]	0.955	0.942	0.06	289.29
|Δ|	0.063		0.075	0.089	0.05	403.65
Sample 2 (*N* = 248)						
Unidimensional	0.158	[0.144, 0.173]	0.906	0.885	0.10	400.33
Three‐dimensional	0.080	[0.064, 0.097]	0.977	0.970	0.05	132.41
|Δ|	0.078		0.071	0.085	0.05	267.92

*Note:* Degrees of freedom = 54 (unidimensional) and 51 (three‐dimensional). χ^2^‐difference tests statistically significant, with *p* < 0.001.

In a next step, we examined the notion of an additional total score. First, we tested a second‐order model empirically, but the model did not converge in both samples, thus, indicating that although the fit is theoretically equivalent to the three‐correlated factor model, the data did not fit well with the model. Finally, we tested the bifactor model with a general factor and three specific factors. Our inspection of the loadings in both samples showed instable loading matrices across samples. For example, Item 8 did not show a loading on the *g*‐factor in Sample 1 (*λ* = 0.08, *p* = 0.296; Sample 2: *λ* = 0.89, *p* < 0.001); Item 3 showed cross‐loadings between Factor I and II in Sample 2 (*λ*s ≥ 0.63), and Items 4 and 12 showed no robust loadings on any specific factor (all loadings available in online supplement B). Taking the evidence together, we rejected the notion of a total score for the German‐language ROCI because (a) the unidimensional model showed clearly stronger misfit in comparison to the three‐factor model, (b) the second‐order model did not converge well in both samples, (c) the bifactor model produced unstable loading patterns, and (d) the three‐factor model provided the best model fit *and* a clear loading structure that is in accordance with the previously established item‐factor assignment.

### 3.2 Measurement Invariance

We examined the invariance of the measurement models of three correlated factors between men and women. Therefore, we aggregated the data from both samples and extracted the data from all men (*n* = 128) and have drawn a random sample of *n* = 128 from the 523 women to ensure equal subsample sizes for testing invariance (Yoon and Lai 2018). All fit indexes are displayed in ESM C. The change indexes did not indicate rejection of (a) the metric model (i.e., constrained loadings; ΔRMSEA = −0.006, ΔCFI = −0.004, and ΔSRMR = 0.012) and (b) the scalar invariance model (ΔRMSEA = −0.003, ΔCFI = 0.003, and ΔSRMR = 0.000), supporting measurement invariance for men and women.

### 3.3 Item Analysis and Reliability

The item difficulties, SDs, and corrected item‐total correlations (CITCs) are displayed in Table 2. As expected, the item difficulties are comparatively high, but there is sufficient variation in responses for each item, thus, suggesting discriminatory power. The latter is also supported by the CITCs (≥ 0.50). The item parameters are stable and replicated across samples. In Samples 1 and 2, 21.3% and 20.2% of participants, respectively, selected the highest or second‐highest response option on at least one‐third of the items. Additionally, 10.3% and 10.5% did so on at least half of the ROCI items. These findings align with Doron et al.'s (2012b) results from nonclinical samples, suggesting that (even when the notion fo a cut‐off is rejected) notable expressions of ROCD are also present in nonclinical German‐speaking populations. In comparison to Doron et al.'s sample, our data were comparable since they did not indicate robust mean differences for Love for Partner (Cohen's *d*_Samples 1/2_ = 0.17/0.19), Relationship Rightness (*d* = 0.17/0.16), and Being Loved by Partner (*d* = 0.03/0.06).

**Table 2:** Item Parameters of the German‐Language ROCI.

	Sample 1 (*N* = 409)				Sample 2 (*N* = 248)			
Item	*M*	SD	CITC	λ	*M*	SD	CITC	λ
Love for Partner (FI)								
6	1.49	0.75	0.67	0.80	1.54	0.91	0.73	0.88
12	1.55	0.82	0.64	0.83	1.50	0.89	0.74	0.90
1	1.56	0.84	0.66	0.80	1.56	0.81	0.63	0.78
8	1.48	0.81	0.78	0.92	1.52	0.87	0.81	0.89
Relationship “Rightness” (FII)								
2	1.66	0.85	0.62	0.85	1.61	0.84	0.67	0.87
7	2.07	1.20	0.51	0.65	1.99	1.17	0.64	0.75
4	2.04	1.05	0.61	0.75	2.09	1.13	0.62	0.74
10	1.75	0.94	0.57	0.74	1.84	1.06	0.68	0.77
Being Loved by Partner (FIII)								
11	1.57	0.93	0.70	0.87	1.56	0.98	0.73	0.85
9	1.61	0.95	0.50	0.53	1.65	1.00	0.60	0.63
5	1.79	0.99	0.69	0.83	1.87	1.06	0.71	0.84
3	1.51	0.85	0.64	0.86	1.53	0.88	0.68	0.91

*Note:* CITC = Corrected item‐total correlation. λ = Item loading from the three‐correlated factors CFA model.

The internal consistencies of the three subscales were comparable to those from other language versions. The α coefficients (ω in brackets) in Samples 1 and 2 were 0.85 (0.85) and 0.87 (0.88) for *Love for Partner*, 0.77 (0.77) and 0.82 (0.82) for *Relationship “Rightness*,*”* and 0.81 (0.82) and 0.84 (0.84) for the *Being Loved by the Partner* scale.

### 3.4 Nomological Validity

Table 3 gives the correlations of the German ROCI with romantic attachment, personality pathology, and relationship satisfaction as well as the measures’ descriptive statistics. The means, SDs, and internal consistencies are comparable to findings from other German‐speaking samples.

**Table 3:** Correlations of the ROCI with Attachment Styles, Personality Pathology, and Relationship Satisfaction.

	Correlations with ROCI			*M*	SD
	Love for Partner	Relationship “Rightness”	Being loved by Partner		
Attachment Styles					
Anxiety	0.34	0.53	0.63	3.39	0.99
Avoidance	0.50	0.40	0.30	2.53	0.85
Relationship satisfaction	−0.37	−0.37	−0.43	3.42	0.47
Happiness in relationship	−0.49	−0.47	−0.34	5.08	1.09
Personality pathology					
Negative affectivity	0.32	0.40	0.40	2.28	0.57
Detachment	0.30	0.25	0.33	1.73	0.50
Antagonism	0.28	0.18	0.25	1.40	0.42
Disinhibition	0.23	0.21	0.34	1.66	0.51
Psychoticism	0.32	0.27	0.34	1.84	0.60

*Note: N* = 248. All correlations statistically significant (*p* < 0.001, two‐tailed). a = Single‐item (no internal consistency measure can be computed).

We found the expected correlations with romantic attachment (*r*s between 0.30 and 0.63), indicating overlap but no redundancy with working models of romantic relationships. Further, the ROCI correlated robustly negatively with relationship satisfaction (*r*s between −0.37 and −0.43) and current levels of happiness with one's relationship (*r*s between −0.34 and −0.49).

Finally, the localization of the ROCI scores in the alternative model of personality pathology showed positive associations with all maladaptive traits, with effect sizes between 0.18 and 0.40. As expected, we found the numerically strongest relationships between the ROCI scales and the domain of negative affectivity (*r*s ≥ 0.32). To control for the interrelations between the maladaptive traits, we computed three regression analyses to identify the contribution of the PID‐5 traits to the variance in each of the ROCI scales (see Supplemental Table D for all coefficients). In short, the *Love for Partner* scores were predicted significantly by negative affectivity (*β* = 0.21, *p* = 0.002) and antagonism (*β* = 0.17, *p* = 0.008), *Relationship “rightness”* was predicted by negative affectivity (*β* = 0.37, *p* < 0.001), and scores of *being loved by the partner* were predicted by negative affectivity (*β* = 0.31, *p* < 0.001; and to a minor degree by detachment, *β* = 0.13, *p* = 0.044). Overall, the three ROCI scales shared between 18% and 24% variance with the broad domains of personality pathology.

### 3.5 Incremental Validity

In line with Doron et al. (2012b), romantic attachment robustly predicted relationship satisfaction negatively (*R* ^2^ = 0.32) and adding the ROCI in Step 2 yielded an increment of 2.2% additional variance (*p* < 0.05). This change translates to a small regression effect size (Δ*f* ^2^ = 0.03). Additionally, we repeated the analyses with the single item of relationship satisfaction: While attachment accounted for 21.1% variance in Step 1, the ROCI accounted for 9.3% incremental variance (*p* < 0.001), which translates to a small‐to‐medium effect size (Δ*f* ^2^ = 0.13). All coefficients from regression analyses are provided in Supplemental Table E.^2^

## 4 Discussion

The present study aimed at examining the German‐language translation of the Relationship Obsessive Compulsive Inventory (ROCI; Doron et al. 2012b) concerning its psychometric properties, factorial structure and measurement invariance between men and women, and external validity. For the latter we replicated findings on romantic attachment and relationship satisfaction and expanded the knowledge of ROCD by localizing ROCI scores in the alternative system of personality pathology.

We examined the factorial structure of the German ROCI by testing four competing models that were derived from the literature. To our knowledge, this was the first study that utilized the WLSMV estimator, which accounts for the response format used in the ROCI (Brauer et al. 2023). In short, our findings showed that the original model proposed by Doron et al. (2012b) with three‐correlated factors was characterized by good model fit in comparison to alternative models and showed a robust loading structure that met the expectations well and replicated across two independent samples. Alternative models representing a total score by means of a general factor of ROCD did either fit the data considerably worse than the three‐factor model (unidimensional model), did not converge (second‐order model), or did not show a replicable and interpretable loading structure (bifactor model). Since the models are comparatively parsimonious, with 12 items assigned to three factors, and our sample sizes meeting the requirements for stable parameter estimation for such models, we do not assume that model complexity or sample size accounts for these issues. Taking these findings together, we did not find evidence for the assumption of a general factor.

We extended our analysis of factorial validity by analyzing measurement invariance of the three‐factor model between men and women. To our knowledge, this was the first research addressing the question whether measurement models differ between men and women and whether comparisons of score distributions between subgroups are affected by measurement. We tested three degrees of invariance and found no evidence for differences in the number of factors (configural invariance), factor loadings (metric invariance), and item intercepts (scalar invariance) between men's and women's measurement models of the ROCI. Accordingly, scores of the German ROCI can be compared well between men and women. This is particularly relevant in research collecting and analyzing dyadic data (e.g., self‐reports of both partners) and testing associations with outcomes such as relationship satisfaction in dyadic analyses using the Actor‐Partner Interdependence Model (Cook and Kenny 2005) and when analyzing partner similarity in ROCD expressions.

Our analyses of the psychometric properties showed that the item difficulties, corrected item‐total correlations, and variances indicated good discriminatory power of all items. Similarly, analyses of internal consistency using two measures (Cronbach's α and McDonald's ω) showed comparable coefficients to earlier research using other language versions of the ROCI (Doron and Derby 2017; Melli et al. 2018). Our results indicated reliability of the scale scores in the range that is expected from brief measures used for research purposes in the field of obsessive‐compulsive disorders (Kemper et al. 2019). When analyzing the response distribution, we found that about 20% of participants endorse a third, and about 10% half of the ROCI items, thus, we conclude in accordance with Doron et al. (2012b) that elevated ROCD expressions can be found in nonclinical populations. Moreover, our response distribution was comparable to the data from Doron et al.'s initial study.

We examined the external validity of the German ROCI in three ways: First, we replicated associations with measures of romantic attachment and relationship satisfaction from prior research (Doron et al. 2012b; Kılıç and Altınok 2021). Overall, prior findings replicated well, showing the expected overlap but no redundancy with attachment and relationship satisfaction. Moreover, the three ROCI scales show differential relations to attachment, with anxiety showing pronounced associations with the *Being Loved by Partner* scale of the ROCI, whereas the *Love for Partner* scale was characterized by avoidance. Thus, our findings speak for the nomological validity of the German ROCI. Secondly, we examined the incremental validity by using Doron et al.'s (2012b) approach to examine the incremental value of the ROCI when predicting relationship satisfaction after controlling for attachment styles. Again, Doron et al.'s findings replicated well when using a global index of relationship satisfaction and a single item of current happiness with one's relationship (Kliem et al. 2012): Regression analyses showed that the ROCI contributes to explain satisfaction even after considering attachment as one of the most robust predictors of satisfaction, thus, speaking for the added value of the ROCI for relationship research. Further, the ROCI explained variance in maladaptive traits beyond insecure attachment. The effect sizes were mostly of small to medium size and future research should provide evidence that statistical significance translates to practical significance. Thirdly, we extended the nomological net of the ROCI and ROCD by embedding the scores in a dimensional system of maladaptive personality traits (Krueger et al. 2012). We found that the ROCI scores correlated positively with maladaptive traits and shared variance with personality pathology without being redundant, thus, speaking for the discriminant validity. In line with Esfahan et al. (2024), our regression analyses showed that predominantly the domain of negative affectivity robustly predicted the ROCI scores. Hence, those with greater ROCD expressions tend to report more negative affect (e.g., sadness and anxiety) and lower emotional regulation (Krueger et al. 2012). In addition, there were scale‐specific relations, as *Love for Partner* showed a minor association with antagonism whereas *Being Loved by the Partner* related also to detachment, showing the differential relations between the ROCI scales and external measures. However, it must be noted that these findings should be interpreted cautiously, as the PID‐5 brief form only assesses very broad domains of personality pathology.

Taking all findings together, we found robust evidence for the good psychometric properties (e.g., item characteristics and scale reliability) and validity (i.e., structural, incremental, and nomological) of the German ROCI. Taking the findings on the factorial structure and the external validity correlations together, we do not recommend computing and interpreting total scores for the German ROCI but instead rely on the three scale scores that describe the facets of *Being Loved by the Partner*, *Relationship “Rightness,” and Love for one's Partner*. While earlier research suggested the existence of a ROCD general factor that translates to a total score, our series of model comparisons did not support this notion for the German‐language version. Accordingly, we recommend that practitioners and researchers interested in cross‐country comparisons using the German ROCI rely on the three scales instead of the total score. This also implies that a previously reported cut‐off score for the Italian version's ROCI total score (Melli et al. 2018) should not be applied for the German version and that norms for the three scales are needed when aiming at individual assessments. In conclusion, we recommend using the German ROCI for research purposes (e.g., cross‐cultural research; testing trainings and interventions; and dyadic studies). Unless further evidence on the cut‐offs is available for the German version, we suggest using the ROCI in clinical settings for purposes that include assessment of individuals cautiously and only supplementary to standard methods such as clinical interviews (see Doron and Derby 2017).

### 4.1 Limitations and Future Directions

Our study has several limitations that should be addressed in future research. First, we relied solely on self‐reports and did not use external criteria (e.g., clinician reports or partner ratings) to examine the validity beyond self‐perceptions and common method variance can play a role (Campbell and Fiske 1959). While our data provide first evidence on the localization of the ROCI facets in domains of maladaptive personality traits, replication and extension using data from a clinical sample is valuable to clarify whether the findings hold in those being externally diagnosed. Future research should extend the validity evidence by testing the agreement between self‐ and partner perceptions to address this issue. Further, the sensitivity of the ROCI scores to change (e.g., in relation to treatment; Doron and Derby 2017; Gorelik et al. 2023) and when predicting outcomes using longitudinal data should be studied to learn more about the predictive validity of the measure. Secondly, we collected data from partnered individuals to ensure that participants have relationship experiences, but using dyadic data and ‐analyses will extend the knowledge of how the ROCI scales relate to outcomes such as relationship satisfaction and attachment in couples. Also, learning more about the role of partner similarity in ROCD when initiating a relationship, during co‐development of partners during their relationship, and whether similarity in lower or higher levels of ROCD might contribute to relationship quality will help understanding ROCD in couples. Also, extension of measurement invariance from persons to time points is desirable, for example, when testing whether the measurement model of the ROCI is invariant among couples at different stages of relationships (e.g., early vs. long‐term relationships) to learn more about how ROCI develops across relationship trajectories. Most participants were women, and the imbalanced gender ratio limits the generalizability of our findings. Thirdly, our reliability analyses were limited to internal consistency, but knowledge about the test‐retest stability is needed to show that the scale scores are relatively stable across time and situations. Fourthly, the selection of our external measures aimed at replicating and extending findings on the nomological net of the ROCI, but our measure of personality pathology was a brief‐form and replication using the full 220‐item measure (Krueger et al. 2012) that also allows to differentiate between facets of personality pathology and the option to also utilize other ratings to increase the validity of ratings is desirable to learn more about the fine‐grained model of the DSM‐5, which allows to derive facet‐level information and learn more about which aspects of pathology go along with ROCD. Moreover, there is robust evidence that the study of maladaptive traits should not rely on self‐reports alone, but also consider other ratings (e.g., by family members) to reduce methodological and perceptual biases (Campbell and Fiske 1959). While our findings extend the nomological net of ROCD, we expect that the use of the ROCI scales in future research will highlight the unique and differential associations between the three facets of ROCD with broad and narrow variables describing individual differences in experiences in relationships (e.g., romantic jealousy; Kılıç and Altınok 2021) and correlates of clinical and nonclinical variables in individuals and couples. Also, the use of the ROCI and the consideration of ROCD might contribute to research on the assumption of the interpersonal core of personality pathology, as discussed by Wright et al. (2022), who recommend recasting personality disorders as interpersonal disorders. Finally, it must be noted that our conclusions about the ROCI and ROCD from this study are based on first data from Germany and should be interpreted cautiously until there is robust evidence supporting the notion of cross‐cultural invariance between the German and original Hebrew language versions.

Further, extension to other variables is valuable. For example, recent research showed the importance of how partners deal with ridicule and being laughed at for relationship quality, attachment, and jealousy (Brauer et al. 2020, 2021), and it would be valuable to examine whether perceptions of ridicule by the partner contribute to understand ROCD. The importance of partner perceptions is also highlighted in the ROCD literature, as symptoms can also present with a focus on the partner, their flaws and doubts about whether they are a good partner (Doron et al. 2012a). To assess the partner‐focused elements of ROCD, a 24‐item self‐report measure is available (Doron et al. 2012a; Melli et al. 2018), but to our knowledge, a German translation is not available. We hope that our findings on the ROCI stimulate researchers to expand the research into the domain of partner‐focused aspects of ROCD to offer a more comprehensive assessment of ROCD in German‐speaking countries beyond relationship‐focused aspects.

## 5 Conclusion

In conclusion, our study provides first evidence that ROCD can be assessed with the ROCI in German‐speaking samples. We hope that our findings on the replication and extension of the structural validity and nomological net of the ROCI and ROCD contribute to the knowledge of the field and stimulate future research on antecedents, consequences, and potential treatment of ROCD, including German‐speaking samples.

## Author Contributions

Study conceptualization, data collection, data preparation, report writing – review: Kay Brauer and Lara Borchardt. Data analysis, report writing draft: Kay Brauer.

## Ethics Statement

This type of research (collection of self‐report data by psychologists for research) is exempt from ethical approval in Germany. The study was performed in accordance with the ethical standards as laid down in the 1964 Declaration of Helsinki and its later amendments and ethical standards outlined by the German Psychological Association.

## Consent

All participants gave informed consent to participate in this study and that their data is used for research purposes.

## Conflicts of Interest

The authors declare no conflicts of interest.

## Open Science

All data and syntaxes to reproduce the analyses are openly available in the Open Science Framework under https://osf.io/gf9yr/.

## Declaration of Generative AI in Scientific Writing

No AI was used in writing the manuscript.

## Supporting information

Electronic Supplementary Material_R1.

## Data Availability

The data that support the findings of this study are openly available in Open Science Framework at https://osf.io/gf9yr/.

## Endnotes

1 Both samples contained one participant at the age of 17 years each (remaining participants ≥ 18 years). We followed the guidelines of the German Association of Psychology and the German Association for Online Research (https://www.dgof.de/wp-content/uploads/2021/01/RL-Minderjaehrigen-neu-2021.pdf) stating that 17‐year‐olds can participate in online studies (exception: product testing) upon their informed consent. All participants were informed that participation in this study was voluntary and anonymous, and that they can quit the online survey at any time.

2 One might also argue that the incremental value of the ROCI when predicting personality pathology after controlling for attachment styles should be clarified. A regression analysis showed that the inclusion of the ROCI explained 4.7% additional variance beyond attachment styles (*R* ^2^ = 0.31) when predicting the PID‐5‐BF total score (see Supplemental Table F for all coefficients). Thus, also showing additional value to predict personality pathology after controlling for attachment.

## References

[jclp70024-bib-0001] Anderson, J. L. , M. Sellbom , and R. T. Salekin . 2018. “Utility of the Personality Inventory for DSM‐5–Brief Form (PID‐5‐BF) in the Measurement of Maladaptive Personality and Psychopathology.” Assessment 25, no. 5: 596–607. 10.1177/1073191116676889. 27827808

[jclp70024-bib-0002] Asparouhov, T. , and B. O. Muthén. 2018. SRMR in Mplus. https://www.statmodel.com/download/SRMR2.pdf.

[jclp70024-bib-0003] Brauer, K. , and R. T. Proyer . 2020. “Gelotophobia in Romantic Life: Replicating Associations With Attachment Styles and Their Mediating Role for Relationship Status.” Journal of Social and Personal Relationships 37, no. 10–11: 2890–2897. 10.1177/0265407520941607.

[jclp70024-bib-0004] Brauer, K. , and R. T. Proyer. 2025. A Study of the Measurement Invariance of the Experiences in Close Relationships (ECR) Questionnaire Across Relationship Status, Romantic Partners, and Gender. *European Journal of Psychological Assessment*. Advance online publication. 10.1027/1015-5759/a000902.

[jclp70024-bib-0005] Brauer, K. , R. T. Proyer , and W. Ruch . 2020. “Extending the Study of Gelotophobia, Gelotophilia, and Katagelasticism in Romantic Life Toward Romantic Attachment.” Journal of Individual Differences 41, no. 2: 86–100. 10.1027/1614-0001/a000307.

[jclp70024-bib-0006] Brauer, K. , J. Ranger , and M. Ziegler . 2023. “Confirmatory Factor Analyses in Psychological Test Adaptation and Development: A Nontechnical Discussion of the WLSMV Estimator.” Psychological Test Adaptation and Development 4, no. 1: 4–12. 10.1027/2698-1866/a000034.

[jclp70024-bib-0007] Brauer, K. , R. Sendatzki , and R. T. Proyer . 2021. “Testing the Associations Between Dispositions Toward Ridicule and Being Laughed at and Romantic Jealousy in Couples: An APIM Analysis.” Journal of Personality 89, no. 5: 883–898. 10.1111/jopy.12621. 33550593

[jclp70024-bib-0008] Brennan, K. A. , C. L. Clark , and P. R. Shaver . 1998. “Self‐Report Measurement of Adult Attachment: An Integrative Overview.” In Attachment Theory and Close Relationships, edited by J. A. Simpson and W. S. Rholes , 46–76. The Guilford Press.

[jclp70024-bib-0009] Campbell, D. T. , and D. W. Fiske . 1959. “Convergent and Discriminant Validation by the Multitrait‐Multimethod Matrix.” Psychological Bulletin 56, no. 2: 81–105. 10.1037/h0046016. 13634291

[jclp70024-bib-0010] Cerea, S. , M. Ghisi , G. Bottesi , E. Carraro , D. Broggio , and G. Doron . 2020. “Reaching Reliable Change Using Short, Daily, Cognitive Training Exercises Delivered on a Mobile Application: The Case of Relationship Obsessive Compulsive Disorder (ROCD) Symptoms and Cognitions in a Subclinical Cohort.” Journal of Affective Disorders 276: 775–787. 10.1016/j.jad.2020.07.043. 32738662

[jclp70024-bib-0011] Chen, F. F. 2007. “Sensitivity of Goodness of Fit Indexes to Lack of Measurement Invariance.” Structural Equation Modeling: A Multidisciplinary Journal 14, no. 3: 464–504. 10.1080/10705510701301834.

[jclp70024-bib-0012] Cohen, J. 1988. Statistical Power Analysis for the Behavioral Sciences, 2nd ed. Lawrence Erlbaum.

[jclp70024-bib-0051] Cook, W. L. , and D. A. Kenny . 2005. “The Actor‐Partner Interdependence Model: A Model of Bidirectional Effects in Developmental Studies.” International Journal of Behavioral Development 29, no. 2: 101–109. 10.1080/01650250444000405.

[jclp70024-bib-0013] Doron, G. , D. Derby , O. Szepsenwol , E. Nahaloni , and R. Moulding . 2016. “Relationship Obsessive–Compulsive Disorder: Interference, Symptoms, and Maladaptive Beliefs.” Frontiers in Psychiatry 7: 182385. 10.3389/fpsyt.2016.00058. PMC4834420 27148087

[jclp70024-bib-0014] Doron, G. , and D. S. Derby . 2017. “Assessment and Treatment of Relationship‐Related OCD Symptoms (ROCD): A Modular Approach.” In The Wiley Handbook of Obsessive Compulsive Disorders, 547–565. Wiley. 10.1002/9781118890233.ch30.

[jclp70024-bib-0015] Doron, G. , D. S. Derby , and O. Szepsenwol . 2014. “Relationship Obsessive Compulsive Disorder (ROCD): A Conceptual Framework.” Journal of Obsessive‐Compulsive and Related Disorders 3, no. 2: 169–180. 10.1016/j.jocrd.2013.12.005.

[jclp70024-bib-0016] Doron, G. , D. S. Derby , O. Szepsenwol , and D. Talmor . 2012a. “Flaws and All: Exploring Partner‐Focused Obsessive‐Compulsive Symptoms.” Journal of Obsessive‐Compulsive and Related Disorders 1, no. 4: 234–243. 10.1016/j.jocrd.2012.05.004.

[jclp70024-bib-0017] Doron, G. , D. S. Derby , O. Szepsenwol , and D. Talmor . 2012b. “Tainted Love: Exploring Relationship‐Centered Obsessive Compulsive Symptoms in Two Non‐Clinical Cohorts.” Journal of Obsessive‐Compulsive and Related Disorders 1, no. 1: 16–24. 10.1016/j.jocrd.2011.11.002.

[jclp70024-bib-0018] Dunn, T. J. , T. Baguley , and V. Brunsden . 2014. “From Alpha to Omega: A Practical Solution to the Pervasive Problem of Internal Consistency Estimation.” British Journal of Psychology 105, no. 3: 399–412. 10.1111/bjop.12046. 24844115

[jclp70024-bib-0019] Esfahan, M. M. , M. N. Ayasrah , F. Ghayoumi , A. Motaharinasab , N. Tayim , and Z. S. P. S. Aghaei . 2024. “The Network Structure of Relationship Obsessive–Compulsive Disorder Presentations: The Interplay Between Rocd Symptoms With Maladaptive and Non‐Maladaptive Personality Traits.” Psychiatric Quarterly 95: 321–339. 10.1007/s11126-024-10079-6. 38896172

[jclp70024-bib-0020] Faul, F. , E. Erdfelder , A. Buchner , and A. G. Lang . 2009. “Statistical Power Analyses Using G* Power 3.1: Tests for Correlation and Regression Analyses.” Behavior Research Methods 41, no. 4: 1149–1160. 10.3758/BRM.41.4.1149. 19897823

[jclp70024-bib-0021] Feeney, J. A. 1999. “Adult Romantic Attachment and Couple Relationships.” In Handbook of Attachment: Theory, Research, and Clinical Applications, edited by J. Cassidy and P. R. Shaver , 355–377. Guilford Press.

[jclp70024-bib-0022] Fernandez, S. , C. Sevil , and R. Moulding . 2021. “Feared Self and Dimensions of Obsessive Compulsive Symptoms: Sexual Orientation‐Obsessions, Relationship Obsessions, and General OCD Symptoms.” Journal of Obsessive‐Compulsive and Related Disorders 28: 100608. 10.1016/j.jocrd.2020.100608.

[jclp70024-bib-0023] Fowler, J. C. , M. A. Patriquin , A. Madan , J. G. Allen , B. C. Frueh , and J. M. Oldham . 2017. “Incremental Validity of the PID‐5 in Relation to the Five Factor Model and Traditional Polythetic Personality Criteria of the DSM‐5.” International Journal of Methods in Psychiatric Research 26, no. 2: e1526. 10.1002/mpr.1526. 27670287 PMC6877239

[jclp70024-bib-0024] Fraley, R. C. 2019. “Attachment in Adulthood: Recent Developments, Emerging Debates, and Future Directions.” Annual Review of Psychology 70: 401–422. 10.1146/annurev-psych-010418-102813. 30609910

[jclp70024-bib-0025] Funder, D. C. , and D. J. Ozer . 2019. “Evaluating Effect Size in Psychological Research: Sense and Nonsense.” Advances in Methods and Practices in Psychological Science 2, no. 2: 156–168. 10.1177/2515245919847202.

[jclp70024-bib-0026] Gomez, R. , S. Watson , and V. Stavropoulos . 2020. “Personality Inventory for DSM–5, Brief Form: Factor Structure, Reliability, and Coefficient of Congruence.” Personality Disorders: Theory, Research, and Treatment 11, no. 1: 69–77. 10.1037/per0000364. 31670543

[jclp70024-bib-0027] Gorelik, M. , O. Szepsenwol , and G. Doron . 2023. “Promoting Couples’ Resilience to Relationship Obsessive Compulsive Disorder (ROCD) Symptoms Using a CBT‐Based Mobile Application: A Randomized Controlled Trial.” Heliyon 9, no. 11: e21673. 10.1016/j.heliyon.2023.e21673. 38027836 PMC10656241

[jclp70024-bib-0028] Hazan, C. , and P. Shaver . 2017. “Romantic Love Conceptualized as an Attachment Process.” In Interpersonal Development, 283–296. Routledge. 10.1037//0022-3514.52.3.511 3572722

[jclp70024-bib-0029] Hu, L. , and P. M. Bentler . 1998. “Fit Indices in Covariance Structure Modeling: Sensitivity to Underparameterized Model Misspecification.” Psychological Methods 3, no. 4: 424–453. 10.1037/1082-989X.3.4.424.

[jclp70024-bib-0030] International Test Commission . 2017. The ITC Guidelines for Translating and Adapting Tests (Second edition). www.InTestCom.org.

[jclp70024-bib-0031] Kemper, C. J. , S. Trapp , N. Kathmann , D. B. Samuel , and M. Ziegler . 2019. “Short Versus Long Scales in Clinical Assessment: Exploring the Trade‐Off Between Resources Saved and Psychometric Quality Lost Using Two Measures of Obsessive–Compulsive Symptoms.” Assessment 26, no. 5: 767–782. 10.1177/1073191118810057. 30501512

[jclp70024-bib-0032] Kliem, S. , A.‐K. Job , C. Kröger , et al. 2012. “Entwicklung und Normierung Einer Kurzform Des Partnerschaftsfragebogens (PFB‐K) An Einer Repräsentativen Deutschen Stichprobe.” Zeitschrift für Klinische Psychologie und Psychotherapie 41, no. 2: 81–89. 10.1026/1616-3443/a000135.

[jclp70024-bib-0033] Körner, R. , and A. Schütz . 2025. “Power Balance and Relationship Quality: An Overstated Link.” Social Psychological and Personality Science 16, no. 5: 471–482. 10.1177/19485506241234391.

[jclp70024-bib-0034] Koster, N. , O. M. Laceulle , P. T. Van der Heijden , et al. 2020. “A Psychometric Evaluation of a Reduced Version of the PID‐5 in Clinical and Non‐Clinical Adolescents.” European Journal of Psychological Assessment 36, no. 5: 758–766. 10.1027/1015-5759/a000552.

[jclp70024-bib-0035] Krueger, R. F. , J. Derringer , K. E. Markon , D. Watson , and A. E. Skodol . 2012. “Initial Construction of a Maladaptive Personality Trait Model and Inventory for DSM‐5.” Psychological Medicine 42, no. 9: 1879–1890. 10.1017/S0033291711002674. 22153017 PMC3413381

[jclp70024-bib-0036] Kılıç, N. , and A. Altınok . 2021. “Obsession and Relationship Satisfaction Through the Lens of Jealousy and Rumination.” Personality and Individual Differences 179: 110959. 10.1016/j.paid.2021.110959.

[jclp70024-bib-0037] Leiner, D. J. 2019. “Too Fast, Too Straight, Too Weird: Non‐Reactive Indicators for Meaningless Data in Internet Surveys.” Survey Research Methods 13, no. 3: 229–248. 10.18148/srm/2019.v13i3.7403.

[jclp70024-bib-0038] Melli, G. , C. Carraresi , A. Pinto , L. Caccico , and E. Micheli . 2018. “Valutare Il Disturbo Ossessivo‐Compulsivo Da Relazione: Proprietà Psicometriche Della Versione Italiana Di Roci E Procsi [Relationship Obsessive–Compulsive Disorder Assessment: The Psychometric Properties of the Italian Version of the ROCI and the PROCSI].” Psicoterapia Cognitiva e Comportamentale 24: 13–37.

[jclp70024-bib-0039] Moshagen, M. , and J. Musch . 2014. “Sample Size Requirements of the Robust Weighted Least Squares Estimator.” Methodology 10, no. 2: 60–70. 10.1027/1614-2241/a000068.

[jclp70024-bib-0040] Muthén, L. K. , and B. O. Muthén . 1997–2019. Mplus 8 User's Manual. Muthén & Muthén.

[jclp70024-bib-0041] Neumann, E. , E. Rohmann , and H. W. Bierhoff . 2007. “Entwicklung und Validierung von Skalen zur Erfassung von Vermeidung und Angst in Partnerschaften.” Diagnostica 53, no. 1: 33–47. 10.1026/0012-1924.53.1.33.

[jclp70024-bib-0042] Reise, S. P. 2012. “The Rediscovery of Bifactor Measurement Models.” Multivariate Behavioral Research 47, no. 5: 667–696. 10.1080/00273171.2012.715555. 24049214 PMC3773879

[jclp70024-bib-0043] Szepsenwol, O. , B. Shahar , and G. Doron . 2016. “Letting It Linger: Exploring the Longitudinal Effects of Relationship‐Related Obsessive‐Compulsive Phenomena.” Journal of Obsessive‐Compulsive and Related Disorders 11: 101–104. 10.1016/j.jocrd.2016.10.001.

[jclp70024-bib-0044] Tinella, L. , L. Lunardi , L. Rigobello , A. Bosco , and F. Mancini . 2023. “Relationship Obsessive Compulsive Disorder (R‐Ocd): The Role of Relationship Duration, Fear of Guilt, and Personality Traits.” Journal of Obsessive‐Compulsive and Related Disorders 37: 100801. 10.1016/j.jocrd.2023.100801.

[jclp70024-bib-0045] Tinella, L. , E. Ricciardi , T. Cosentino , et al. 2024. “Deontological Guilt Mediates the Effects of Personality on the Symptoms of Romantic Relationship Obsessive Compulsive Disorder (ROCD).” Clinical Neuropsychiatry 21, no. 3: 205–216. 10.36131/cnfioritieditore20240306. 38988679 PMC11231728

[jclp70024-bib-0046] Trak, E. , and M. İnözü . 2017. “A New Obsessive‐compulsive Symptom Type: The Psychometric Properties of the Turkish Formsforms of Relationship Obsessions and Compulsions Inventory and Partner‐related Obsessive‐Compulsive Symptoms Inventory.” Journal of Clinical Psychiatry 20, no. 3: 171–185. 10.5505/kpd.2017.93063.

[jclp70024-bib-0047] Wright, A. G. C. , W. R. Ringwald , C. J. Hopwood , and A. L. Pincus . 2022. “It's Time to Replace the Personality Disorders With the Interpersonal Disorders.” American Psychologist 77, no. 9: 1085–1099. 10.1037/amp0001087. 36595407

[jclp70024-bib-0048] Yoon, M. , and M. H. C. Lai . 2018. “Testing Factorial Invariance With Unbalanced Samples.” Structural Equation Modeling: A Multidisciplinary Journal 25, no. 2: 201–213. 10.1080/10705511.2017.1387859.

[jclp70024-bib-0049] Zijlmans, E. A. O. , J. Tijmstra , L. A. van der Ark , and K. Sijtsma . 2018. “Item‐Score Reliability in Empirical‐Data Sets and Its Relationship With Other Item Indices.” Educational and Psychological Measurement 78, no. 6: 998–1020. 10.1177/0013164417728358. 30542214 PMC6236637

[jclp70024-bib-0050] Zimmermann, J. , D. Altenstein , T. Krieger , et al. 2014. “The Structure and Correlates of Self‐Reported DSM‐5 Maladaptive Personality Traits: Findings From Two German‐Speaking Samples.” Journal of Personality Disorders 28, no. 4: 518–540. 10.1521/pedi_2014_28_130. 24511899

