# Supplementary material for: Validation of the German Relationship‐Obsessive Compulsive Inventory: Testing the Factorial Structure, Measurement Invariance, and External Validity
Source: J Clin Psychol. 2025 Aug 8;81(11):1143–54. doi: 10.1002/jclp.70024 (PMC12501827; doi:10.1002/jclp.70024)
Supplement: Supplementary file 1 — Electronic Supplementary Material_R1. [file JCLP-81-1143-s001.docx]

**Electronic Supplementary Material**

**Supplemental Table A**

*Items of the German-language Relationship Obsessive Compulsive Inventory (ROCI)*

| Item | Wording |
| --- | --- |
| 1 | Der Gedanke, dass ich meinen Partner/meine Partnerin nicht wirklich liebe, verfolgt mich |
| 2 | Ich zweifle ständig an meiner Beziehung |
| 3 | Ich zweifle häufig an der Liebe meines Partners/meiner Partnerin |
| 4 | Ich überprüfe ständig, ob sich meine Beziehung “richtig” anfühlt |
| 5 | Ich suche ständig nach Beweisen dafür, dass mein Partner/meine Partnerin mich wirklich liebt |
| 6 | Ich habe das Gefühl, dass ich mich immer wieder daran erinnern muss, warum ich meinen Partner/meine Partnerin liebe |
| 7 | Der Gedanke, dass in meiner Beziehung etwas “nicht stimmt“, beunruhigt mich extrem |
| 8 | Ich zweifle ständig an meiner Liebe zu meinem Partner/meiner Partnerin |
| 9 | Ich frage meinen Partner/meine Partnerin ständig, ob er/sie mich wirklich liebt |
| 10 | Ich suche häufig die Bestätigung, dass meine Beziehung “richtig” ist |
| 11 | Ich mache mir ständig Sorgen, dass mein Partner/meine Partnerin nicht wirklich mit mir zusammen sein will |
| 12 | Ich habe den Drang, immer wieder zu überprüfen, wie sehr ich meinen Partner/meine Partnerin liebe |

*Note*. Response options are 1 = *trifft gar nicht zu*, 5 = *trifft sehr zu*.

**Supplemental Table B**

*Loadings from Bifactor Models Analyses with a General Factor (G) and Three Scale-Specific Factors (I, II, and III)*

|  | Sample 1 (*N* = 409) | | | |  | Sample 2 (*N* = 248) | | | |
| --- | --- | --- | --- | --- | --- | --- | --- | --- | --- |
| Item | G | I | II | III |  | G | I | II | III |
| 1 | .75 | .11 | -.25 | -.01 |  | .72 | .03 | .23 | -.29 |
| 2 | .84 | .20 | -.01 | -.14 |  | .86 | -.03 | .20 | -.01 |
| 3 | .44 | .78 | -.03 | -.25 |  | .55 | .63 | .71 | -.01 |
| 4 | .75 | .17 | .35 | .02 |  | .73 | .09 | .02 | .13 |
| 5 | .40 | .67 | .35 | .22 |  | .49 | .70 | .00 | .21 |
| 6 | .81 | -.10 | -.07 | .08 |  | .88 | -.13 | -.05 | -.05 |
| 7 | .52 | .45 | .01 | -.03 |  | .74 | .07 | .06 | .04 |
| 8 | .93 | -.04 | -.33 | -.02 |  | .89 | -.13 | .00 | -.37 |
| 9 | .08 | .64 | .02 | .49 |  | .30 | .69 | -.14 | .06 |
| 10 | .63 | .34 | .26 | .29 |  | .77 | .12 | -.05 | .41 |
| 11 | .41 | .78 | -.02 | -.02 |  | .49 | .68 | .11 | -.04 |
| 12 | .81 | .02 | .04 | .36 |  | .89 | .07 | -.15 | .03 |

**Supplemental Table C**

*Intercorrelations Between External Study Measures*

|  | (1) | (2) | (3) | (4) | (5) | (6) | (7) | (8) |
| --- | --- | --- | --- | --- | --- | --- | --- | --- |
| PID-5 |  |  |  |  |  |  |  |  |
| (1) Negative Affect |  |  |  |  |  |  |  |  |
| (2) Detachment | .38 |  |  |  |  |  |  |  |
| (3) Antagonism | .12 | .30 |  |  |  |  |  |  |
| (4) Disinhibition | .28 | .31 | .35 |  |  |  |  |  |
| (5) Psychoticism | .45 | .53 | .33 | .48 |  |  |  |  |
| Attachment |  |  |  |  |  |  |  |  |
| (6) Anxiety | .61 | .18 | .23 | .26 | .29 |  |  |  |
| (7) Avoidance | .25 | .49 | .49 | .25 | .33 | .25 |  |  |
| Satisfaction |  |  |  |  |  |  |  |  |
| (8) Single Item | -.11 | -.15 | -.07 | -.15 | -.13 | -.27 | -.43 |  |
| (9) Total | -.18 | -.25 | -.09 | -.20 | -.19 | -.43 | -.47 | .52 |

*Note*. *N* = 248.

**Supplemental Table D**

*Regression Analyses Predicting ROCI Scales by PID-5-BF Domain Scores*

|  | Love for Partner | | |  | Relationship Rightness | | |  | Being Loved by Partner | | |
| --- | --- | --- | --- | --- | --- | --- | --- | --- | --- | --- | --- |
|  | *b* | β | *p* |  | *b* | β | *p* |  | *b* | β | *p* |
| Negative Affect | 0.27 | .21 | .002 |  | 0.55 | .37 | <.001 |  | 0.43 | .31 | <.001 |
| Detachment | 0.14 | .09 | .194 |  | 0.06 | .03 | .636 |  | 0.15 | .09 | .190 |
| Antagonism | 0.30 | .17 | .008 |  | 0.16 | .08 | .231 |  | 0.17 | .09 | .162 |
| Disinhibition | 0.03 | .02 | .746 |  | 0.03 | .02 | .764 |  | 0.21 | .13 | .044 |
| Psychoticism | 0.14 | .12 | .144 |  | 0.07 | .05 | .520 |  | 0.08 | .06 | .436 |
| *R*^2^ | .18 | | |  | .19 | | |  | .24 | | |

*Note*. *N* = 248.

**Supplemental Table E**

*Stepwise Regression Analyses Testing the Incremental Validity of the ROCI Scales Beyond ECR When Predicting Relationship Satisfaction*

|  | RQQ Total Score | | | |  | Single Item | | | |
| --- | --- | --- | --- | --- | --- | --- | --- | --- | --- |
|  | *b* | β | *p* | Δ*R*^2^ |  | *b* | β | *p* | Δ*R*^2^ |
| Step 1 |  |  |  | .32 |  |  |  |  | .21 |
| Anxiety | -0.21 | -.38 | <.001 |  |  | -0.49 | -.38 | <.001 |  |
| Avoidance | -0.16 | -.33 | <.001 |  |  | -0.20 | -.18 | .002 |  |
| Step 2 |  |  |  | .02 |  |  |  |  | .10 |
| Anxiety | -0.18 | -.33 | <.001 |  |  | -0.29 | -.23 | <.001 |  |
| Avoidance | -0.11 | -.22 | .002 |  |  | -0.02 | -.02 | .822 |  |
| Love for Partner | -0.04 | -.07 | .446 |  |  | -0.35 | -.24 | .010 |  |
| Relationship Rightness | 0.02 | .03 | .744 |  |  | -0.15 | -.12 | .225 |  |
| Being Loved by Partner | -0.10 | -.18 | .013 |  |  | -0.12 | -.09 | .215 |  |

*Note*. *N* = 248.

**Supplemental Table F**

*Stepwise Regression Analyses Testing the Incremental Validity of the ROCI Scales Beyond ECR When Predicting Personality Pathology (PID-5-BF Total Score)*

|  | *b* | β | *p* | Δ*R*^2^ |
| --- | --- | --- | --- | --- |
| Step 1 |  |  |  | .31 |
| Anxiety | 0.14 | .38 | <.001 |  |
| Avoidance | 0.14 | .33 | <.001 |  |
| Step 2 |  |  |  | .05 |
| Anxiety | 0.10 | .28 | <.001 |  |
| Avoidance | 0.10 | .24 | <.001 |  |
| Love for Partner | 0.12 | .24 | .008 |  |
| Relationship Rightness | -0.07 | -.17 | .068 |  |
| Being Loved by Partner | 0.09 | .20 | .004 |  |

*Note*. *N* = 248.
